# Combined endocardial and epicardial ablation of drug-refractory ventricular tachycardia by direct ventricular puncture

**DOI:** 10.1016/j.hrcr.2021.09.006

**Published:** 2021-09-17

**Authors:** Hasan Ashraf, Nareg Minaskeian, Kristen Sell-Dotin, Hicham Z. El Masry

**Affiliations:** Mayo Clinic, Phoenix, Arizona

**Keywords:** Ventricular tachycardia, Epicardial and endocardial ablation, Mechanical aortic and mitral valves, Thoracotomy, Surgical cryoablation, Transapical access

## Introduction

Mechanical heart valves in both aortic and mitral positions pose a significant challenge for left ventricular (LV) access during ventricular tachycardia (VT) ablation using the standard antegrade transseptal or the retrograde transaortic approach. The customary approaches are not possible because of risk of catheter entrapment and death, and alternative strategies are necessary. We describe a variation of the transapical LV access via direct surgical visualization in which we successfully utilized both endocardial and epicardial ablation in a patient with drug-refractory VT.

## Case report

A 57-year-old male patient with ischemic cardiomyopathy (LV ejection fraction 25%) and recurrent episodes of VT was evaluated by the heart rhythm team. He has a complex past medical history, including Hodgkin lymphoma during childhood treated with chest radiation, leading to early development of coronary artery disease, and inferior myocardial infarction treated with proximal right coronary artery stenting at the age of 36. He eventually developed worsening coronary artery disease and underwent triple vessel coronary artery bypass surgery 2 years later. Following that, he developed calcific valvular heart disease and severe cardiomyopathy and underwent mechanical mitral and aortic valve replacement (2011), then a dual-chamber implantable cardioverter-defibrillator (ICD) implantation for primary prevention in 2012. On follow-up, he developed recurrent episodes of VT appropriately treated with ICD shocks, which proved refractory to antiarrhythmic therapy including sotalol, mexiletine, and amiodarone.

After multidisciplinary consultation, he was deemed not a candidate for heart transplant owing to hostile chest and a porcelain aorta, nor a candidate for standard approach of endocardial ablation owing to mechanical mitral and aortic valves. We initially attempted an epicardial ablation using a subxiphoid approach; however, significant amounts of adhesions prevented advancement of the guidewire in the pericardium. We also attempted transinterventricular septal access but, despite successful access to the LV, we were unable to advance the sheath through a hypertrophied septum and the procedure was aborted at that point.

**Figure 1:**
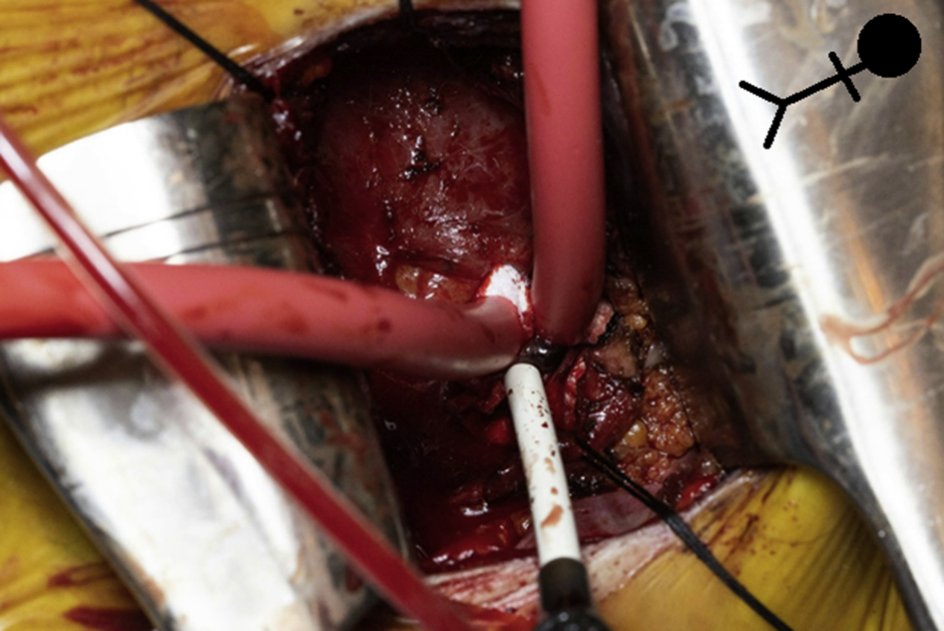
Exposure of the lateral apex of the left ventricle. A 9F sheath can be seen at the access site, along with the purse-string sutures, which were used to obtain hemostasis at the termination of the case. Stick figure for patient position reference.

The patient continued to worsen clinically, with frequent episodes of VT and ICD shocks despite titrating his oral amiodarone dose to 400 mg twice daily. One last attempt at ablation was planned using a combined transapical endocardial and epicardial approach. The procedure was performed under general anesthesia with transesophageal echocardiographic visualization of the heart. Standard surgical anticoagulation protocol was followed with the initiation of heparin drip preprocedurally, with planned heparin bridging and resumption of warfarin on the night after the procedure, with a goal international normalized ratio of 2.5–3.5. Standard femoral venous access was obtained, including a 6F deflectable quadripolar catheter, which was placed in the right ventricle, and a 10F CartoSound (Biosense Webster Inc, Irvine, CA) intracardiac echocardiography probe in the right heart. Subsequently, a mini–left thoracotomy was performed using a 6-cm incision along the fifth intercostal space, exposing the cardiac apex. The pericardium was opened using electrocautery and careful dissection was performed to take down the pericardial adhesions. The LV apex was identified and an appropriate cannulation site, around the inferolateral apex, was confirmed with transesophageal echocardiographic guidance. Two 3-0 Ethibond pledgeted sutures were used to create purse strings on the LV apex, and the apex was accessed under direct visualization with a Cook (Cook Medical Inc, Bloomington, IN) needle and wire. The tract was dilated, and a standard 9F Cordis sheath was inserted into the access site, through which an 8F irrigated bidirectional ThermoCool (Biosense Webster Inc, Irvine CA) ablation catheter was inserted (Figure 1). Unfractionated heparin was initiated upon access of the left ventricle and continued throughout the procedure, with a target activated clotting time of 250–300 seconds. The procedure was performed in a hybrid electrophysiology lab with the standard electrophysiology fluoroscopy settings. During ablation, frequent fluoroscopic evaluation in the right anterior oblique projection and continuous intracardiac monitoring were performed to avoid catheter entanglement in the mechanical valves.

**Figure 2:**
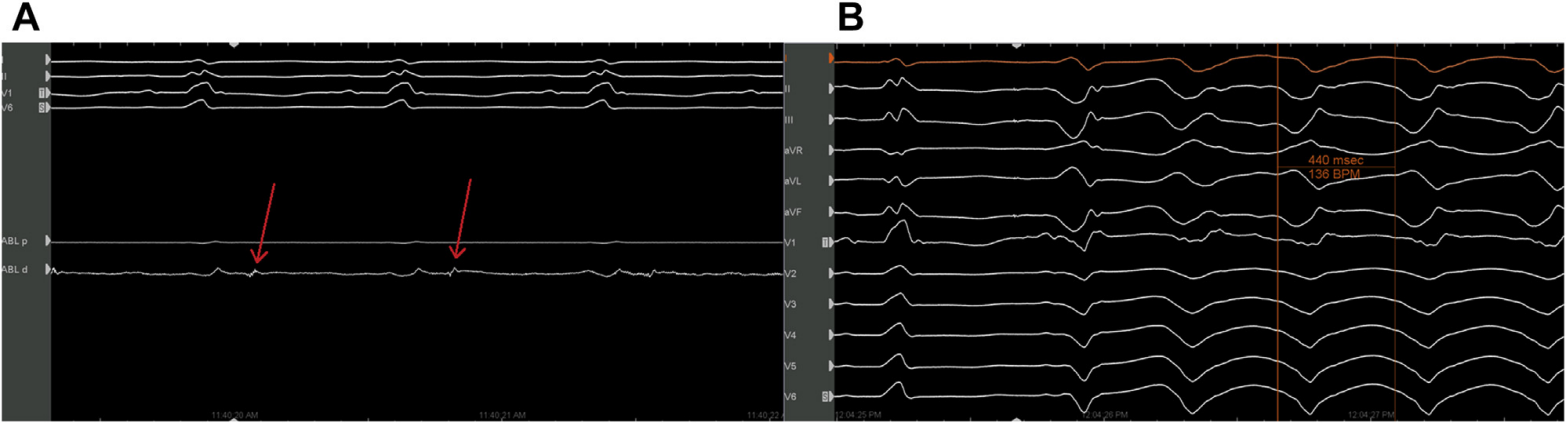
**A:** Example of late potentials during sinus rhythm that were marked and ablated. **B:** Hemodynamically unstable ventricular tachycardia (cycle length 440 ms) requiring cardioversion restoring sinus rhythm.

**Figure 3:**
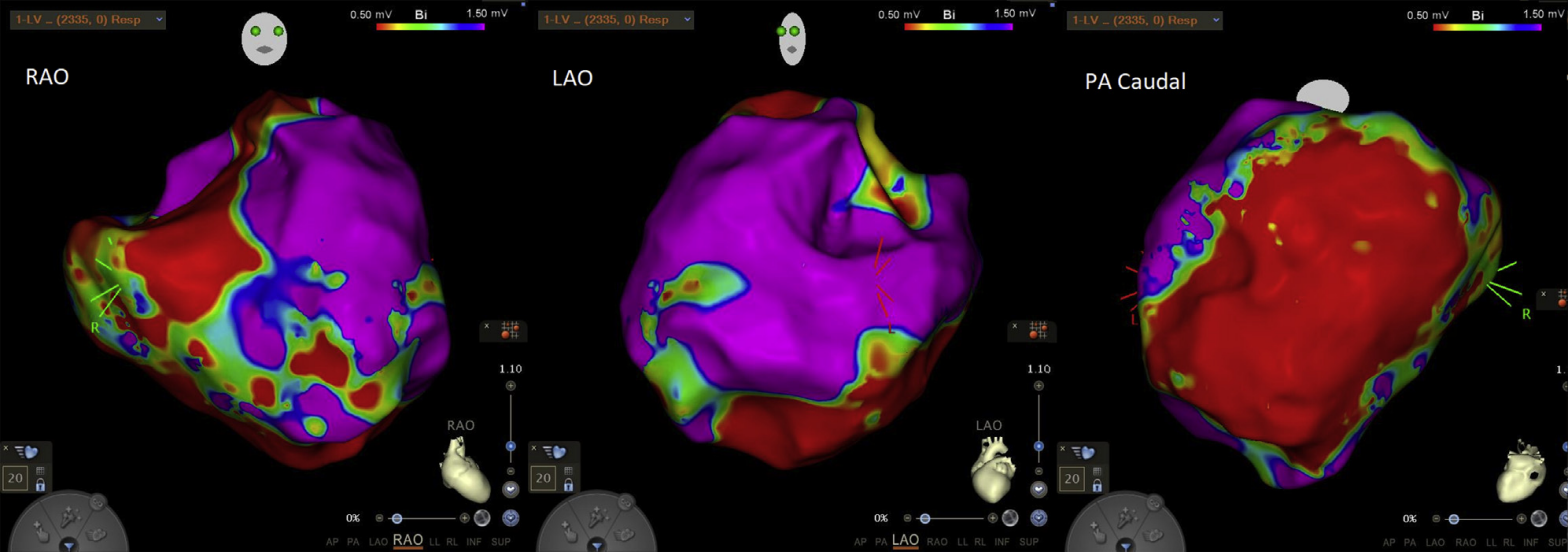
Voltage map showing dense inferior wall scar.

Electroanatomical mapping was performed using Carto software (Biosense Webster Inc, Irvine, CA) recording voltage and marking local abnormal ventricular activations (Figure 2A). The voltage map revealed a dense inferior wall scar extending from the apex to the base (Figure 3), and local abnormal ventricular activations were tagged and concomitantly ablated. Of note, hemodynamically unstable VT (cycle length 440 ms, Figure 2B) was induced by catheter manipulation, requiring external cardioversion restoring sinus rhythm, and we were unable to perform mapping and entrainment during VT.

After completion of endocardial ablation from the transapical site, attention was turned to the epicardium, particularly the apex, which was incompletely mapped and ablated endocardially around the sheath entry site owing to technical difficulty. An Articure (Atricure Inc, Mason, OH) cryoablation probe was used to apply linear lesions along the entire apex, including inferior, lateral, and septal segments. Epicardial mapping was not performed owing to the technical mapping limitations in an open chest. Endpoints were assessed with an aggressive programmed ventricular stimulation protocol using a drive train of 600 ms with up to 3 extrastimuli to ventricular refractoriness. No sustained ventricular arrhythmias were inducible, and this was deemed a successful procedural endpoint.

He was transferred to the intensive care unit and progressed well postoperatively, without any complications. Oral warfarin with a heparin bridge was initiated the same night of the procedure. He was extubated the following day and discharged 2 days later without antiarrhythmic medication. On 2-month follow-up, he remains physically active and device interrogation revealed no ventricular arrhythmias (Supplemental Figure S1).

## Discussion

Patients with drug-refractory VT and double mechanical valve prostheses present a unique challenge in that conventional percutaneous LV access via antegrade transseptal or retrograde aortic approach is not possible (no-entry LV). A number of these VTs can be ablated from the right ventricle, such as with bundle branch reentry, or with epicardial access.^1^ However, patients with surgically implanted prostheses may have pericardial adhesions that preclude epicardial ablation. Additionally, the epicardial approach may not be optimal given the endocardial location of reentrant circuits, which occurs commonly in ischemic cardiomyopathies, which may decrease the efficacy of isolated epicardial ablations that do not extend deep enough to the endocardial surface to alter the arrhythmogenic substrate.

Alternative approaches to these challenging patients have been described, including transcoronary ethanol ablation, surgical cryoablation, percutaneous trans–right atrial access to the LV through the creation of an iatrogenic Gerbode defect at the inferior-septal process of the LV, and interventricular septal puncture.^2^, ^3^, ^4^, ^5^, ^6^ However, these approaches are technically challenging and success is highly dependent on specific anatomy, which can vary considerably between patients. Radiation therapy has also been used for VT ablation, but long-term follow-up is limited and complications are not insignificant.^7^ This case demonstrates the major advantages of an open surgical approach, which includes the ability to deliver both endocardial and epicardial lesions, significantly increasing the chance of success. An additional advantage is the theoretical feasibility for most LV VTs, as the apical approach would allow for easy catheter maneuverability and access of most of the LV. Using a deflectable ablation catheter, we were able to access most of the LV with minimal difficulty even without a deflectable sheath, except for the location in immediate proximity of the catheter access site. Because the LV apex is easily accessible with a minithoracotomy and is thinner in most patients compared to other, more basal LV walls, it provides an ideal anatomy for epicardial cryoablation. This permits transmural application of ablative lesions for substrate modification, and thus increases chance of success, as demonstrated in our patient.

A prior case series that utilized both percutaneous transapical LV access and surgical access noted a significant drawback with surgical access because of weak tissue support from the LV myocardium, which could lead to sheath dislodgement.^8^ This is indeed a possibility, though in our case we stabilized the sheath manually throughout the case, rendering the risk of sheath dislodgement negligible. Although this may be cumbersome, we believe that this is far outweighed by the risks and high incidence of adverse outcomes of a percutaneous approach.

The major drawback of a more invasive approach is mitigated by the relative technical straightforwardness of the procedure, its general applicability to most LV VTs, the high likelihood of success, and the low incidence of complications. Although experience is limited in these complex ablations, prior cases have suggested that most risks with transapical approaches are due to the percutaneous approach rather than the surgical one. A minithoracotomy approach would also permit better control of hemostasis and fewer complications, and is not necessarily associated with a lengthier hospital stay. Concerns with the surgical approach include holding anticoagulation for mechanical valves, though this can be accomplished within just a few hours if adequate bridging is accomplished; and with newer, less thrombogenic mechanical valves, the concern for thromboembolism is significantly diminished. Additionally, we demonstrated that with careful selection of patients and with utilization of good surgical technique, speedy discharge within a few days is feasible. Though further data is undeniably needed regarding long-term outcomes and comparative outcomes between different strategies for VT ablation, this case adds to the growing literature regarding the relative technical ease, feasibility, excellent complication profile, and efficacy of this approach.

**Key Teaching Points**

• No-access left ventricles with mechanical aortic and mitral valves pose a challenge for drug-refractory ventricular tachycardia ablation. A combined surgical approach in these patients is safe and feasible.
• A combined surgical approach with minithoracotomy and epicardial and endocardial ablation results in adequate and efficacious lesion formation.
• Recovery time, patient and procedural safety, and outcomes are favorable, without the need for prolonged hospitalization.

## Appendix Supplementary data

Supplemental Figure S1 Cardiac compass from the patient’s ICD interrogation superimposed to the timeline of ablations and medical therapy. Escalation of amiodarone therapy and addition of mexiletine was performed after the first ablation attempt. Further titration of the amiodarone dose (400 mg twice daily) and multiple oral reloading was attempted prior to the second ablation. Antiarrhythmics were stopped immediately after the hybrid ablation without recurrence to date.
