# Supplementary material for: Combined endocardial and epicardial ablation of drug-refractory ventricular tachycardia by direct ventricular puncture
Source: HeartRhythm Case Rep. 2021 Sep 17;7(12):840–3. doi: 10.1016/j.hrcr.2021.09.006 (PMC8695292; doi:10.1016/j.hrcr.2021.09.006)
Supplement: Supplemental Figure S1 — Cardiac compass from the patient’s ICD interrogation superimposed to the timeline of ablations and medical therapy. Escalation of amiodarone therapy and addition of mexiletine was performed after the first ablation attempt. Further titration of the amiodarone dose (400 mg twice daily) and multiple oral reloading was attempted prior to the second ablation. Antiarrhythmics were stopped immediately after the hybrid ablation without recurrence to date. [file mmc1.docx]

Supplement S1


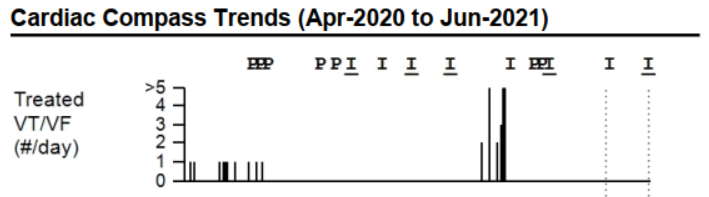

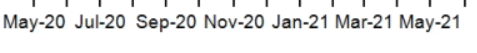


^First VT ablation attempt^

^Hybrid VT ablation^

Amiodarone

Mexiletine

Supplement S1: Cardiac compass from the patient’s ICD interrogation superimposed to the timeline of ablations and medical therapy. Escalation of amiodarone therapy and addition of mexiletine was performed after the first ablation attempt. Further titration of the amiodarone dose (400 mg twice daily) and multiple oral reloading was attempted prior to the second ablation. Antiarrhythmics were stopped immediately after the hybrid ablation without recurrence to date.
